# Diagnostic Criteria for Cancer‐Associated Cachexia: Insights from a Multicentre Cohort Study

**DOI:** 10.1002/jcsm.13703

**Published:** 2025-02-13

**Authors:** Zhenyu Huo, Feifei Chong, Na Li, Siyu Luo, Liangyu Yin, Jie Liu, Mengyuan Zhang, Jing Guo, Yang Fan, Ling Zhang, Xin Lin, Hongmei Zhang, Muli Shi, Xiumei He, Zongliang Lu, Ning Tong, Wei Li, Jiuwei Cui, Zengqing Guo, Qinghua Yao, Fuxiang Zhou, Ming Liu, Zhikang Chen, Huiqing Yu, Minghua Cong, Tao Li, Zengning Li, Pingping Jia, Min Weng, Chunhua Song, Hanping Shi, Hongxia Xu, The Investigation on Nutrition Status and Clinical Outcome of Common Cancers (INSCOC) Group

**Affiliations:** ^1^ Department of Clinical Nutrition, Daping Hospital Army Medical University (Third Military Medical University) Chongqing China; ^2^ Chongqing Municipal Health Commission Key Laboratory of Intelligent Clinical Nutrition and Transformation Chongqing China; ^3^ Department of Nephrology, the Key Laboratory for the Prevention and Treatment of Chronic Kidney Disease of Chongqing, Chongqing Clinical Research Center of Kidney and Urology Diseases, Xinqiao Hospital Army Medical University (Third Military Medical University) Chongqing China; ^4^ Department of Clinical Nutrition The Thirteenth People's Hospital of Chongqing Chongqing China; ^5^ Department of Clinical Nutrition Chongqing University Jiangjin Hospital Chongqing China; ^6^ Cancer Center The First Hospital of Jilin University Jilin China; ^7^ Department of Medical Oncology, Fujian Cancer Hospital Fujian Medical University Cancer Hospital Fuzhou Fujian China; ^8^ Department of Integrated Chinese and Western Medicine Cancer Hospital of the University of Chinese Academy of Science (Zhejiang Cancer Hospital) Hangzhou Zhejiang China; ^9^ Department of Oncology Zhongnan Hospital of Wuhan University Wuhan Hubei China; ^10^ Department of Colorectal Surgery The Fourth Affiliated Hospital of Harbin Medical University Harbin Heilongjiang China; ^11^ Department of Colorectal and Anal Surgery Xiangya Hospital of Central South University Changsha Hunan China; ^12^ Department of Palliative Care and Department of Geriatric Oncology Chongqing University Cancer Hospital Chongqing China; ^13^ Comprehensive Oncology Department, National Cancer Center/Cancer Hospital Chinese Academy of Medical Sciences and Peking Union Medical College Beijing China; ^14^ Department of Radiation Oncology, Sichuan Cancer Hospital and Institute, Sichuan Cancer Center, School of Medicine University of Electronic Science and Technology of China Chengdu Sichuan China; ^15^ Department of Clinical Nutrition The First Hospital of Hebei Medical University Shijiazhuang Hebei China; ^16^ Department of Gastrointestinal Surgery and Department of Clinical Nutrition, Beijing Shijitan Hospital Capital Medical University Beijing China; ^17^ Department of Clinical Nutrition The First Affiliated Hospital of Kunming Medical University Kunming Yunnan China; ^18^ Department of Epidemiology, College of Public Health Zhengzhou University Zhengzhou Henan China; ^19^ Key Laboratory of Cancer FSMP for State Market Regulation Beijing China

**Keywords:** cancer cachexia, Fearon, mortality, muscle mass, neutrophil‐to‐lymphocyte ratio

## Abstract

**Background:**

To explore the association between cachexia, as defined by different diagnostic criteria, and the risk of mortality in individuals with cancer. We also examined which diagnostic criteria are more feasible and appropriate for cancer‐associated cachexia in clinical practice.

**Methods:**

A multicentre cohort study was conducted, which involved 5769 participants with cancer. The diagnosis of cachexia was made by applying the initial Fearon criteria (with the appendicular skeletal muscle mass index [ASMI]) and six modified criteria: (1) evaluating the muscle mass through the mid‐upper‐arm muscle area (MAMA), (2) fat‐free mass index (FFMI), (3) calf circumference (CC), (4) hand grip strength (HGS), (5) neutrophil‐to‐lymphocyte ratio (NLR) and (6) omission of reduced muscle mass. The correlations between cancer cachexia diagnosed by different definitions and survival were assessed using Kaplan–Meier analyses and multivariable‐adjusted Cox models. The sensitivity, specificity, positive likelihood ratios, negative likelihood ratios, AUC value, Youden index and weighted kappa coefficient were calculated for each set of criteria.

**Results:**

The final analysis included 5110 patients diagnosed with 15 different types of cancer, with a median age of 56. Out of these, 2490 (48.7%) were male. The prevalence of cancer cachexia based on the Fearon criteria was 26.5%, ranging from 21.8% to 32.2% with the six modified criteria. Following adjustment for age, sex, clinical stage and cancer site, cachexia defined by Fearon criteria was associated with a noteworthy increase in mortality (HR, 1.275; 95% CI, 1.136–1.430; *p* < 0.001), ranging from 1.237 (95% CI, 1.106–1.383; *p* < 0.001) to 1.382 (95% CI, 1.226–1.557; *p* < 0.001) by the six modified criteria. All six modified criteria presented adequate performance indicators (all *p* < 0.001), with sensitivity ranging from 82.4% (95% CI, 80.2%–84.3%) to 90.7% (95% CI, 89.0%–92.2%), specificity ranging from 86.9% (95% CI, 85.7%–87.9%) to 100.0% (95% CI, 99.9%–100.0%) and AUC ranging from 0.860 (95% CI, 0.850–0.869) to 0.932 (95% CI, 0.925–0.939). The modified criteria also showed strong (Fearon criteria with NLR: *κ* = 0.673, 95% CI, 0.651–0.695) to almost perfect (Fearon criteria without reduced muscle mass [RMM]: *κ* = 0.873, 95% CI, 0.857–0.888) consistency with the original Fearon criteria.

**Conclusions:**

Cachexia defined by the Fearon criteria and the six modified criteria can predict the survival of cancer patients. All criteria provided a precise diagnosis and were feasible to use in clinical settings.

## 1 Introduction

Cancer‐associated cachexia is a complicated disorder with multiple factors that primarily lead to weight loss, specifically in skeletal muscle and adipose tissue, often accompanied by systemic inflammation [1]. The prevalence of cachexia varies depending on the type of cancer, ranging from 35% to 80%, with higher rates observed in patients with pancreatic and gastric cancers [2, 3]. Cachexia has detrimental effects on cancer patients' nutrition, quality of life and the effectiveness of anticancer chemotherapy [4]. Conventional nutritional support and other treatments are insufficient to fully reverse cachexia, resulting in gradual functional decline and increased cancer mortality [4, 5, 6]. Previous studies suggest that 20%–30% of cancer‐related deaths can be attributed to cachexia [7]. Therefore, it is crucial to actively prevent, diagnose and treat cachexia to minimize its negative impact on patient outcomes [8].

In 2011, Fearon et al. conducted an international Delphi consensus to establish a specific definition and conceptual framework for cancer‐related cachexia [6]. The diagnosis of cachexia can be made based on three proposed criteria: (1) weight loss of more than 5% within the last 6 months, (2) weight loss of more than 2% plus a low body mass index (BMI) and (3) weight loss of more than 2% plus reduced muscle mass (RMM). Despite the recommendation that patients should regularly be evaluated for muscle mass and strength, there was a lack of agreement on the specific approach to be used [9]. Fearon et al. recommended using the appendicular skeletal muscle mass index (ASMI), which is measured by cross‐sectional imaging (magnetic resonance imaging [MRI] or computed axial tomography [CT]), dual energy X‐ray imaging (DEXA) and/or bioimpedance analysis (BIA) for muscle mass assessment [6, 10]. In practice, these techniques are not yet standardized, are often inaccessible for research studies and are not practical in the majority of health care settings [11]. Therefore, it is necessary to find simple, valid, reliable and inexpensive criteria to replace the ASMI in muscle mass assessment for the diagnosis of cancer‐associated cachexia.

According to Fearon et al., the mid‐upper‐arm muscle area (MAMA) which can be obtained through anthropometry can also be used to assess whether there is an RMM, with recommended cut‐off values for males of < 32 cm^2^ or females of < 18 cm^2^ [6]. Previous research has indicated that patients with head and neck cancer with a low MAMA have a higher risk of poor survival, regardless of their inflammatory status and weight loss [12]. The fat‐free mass index (FFMI) was also recommended to define pulmonary cachexia in a statement from the European Respiratory Society [13] and has been shown to significantly predict mortality in patients with chronic obstructive pulmonary disease [14]. The calf circumference (CC) has been proposed as a surrogate marker of muscle mass when imaging technologies are not available [15], and the Asian Working Group for Sarcopenia (AWGS) 2019 consensus recommended that the CC can be used to predict sarcopenia or a low skeletal muscle mass [16]. Furthermore, the hand grip strength (HGS) is also a useful alternative to measure the muscle mass. Several previous studies indicated that a low HGS was linked with increased all‐cause mortality in patients with cancer cachexia [17, 18]. Recently, the neutrophil‐to‐lymphocyte ratio (NLR), as a measure of systemic inflammation, has also been attracting interest as an indicator of cachexia [19]. In a study involving multiple medical centres in China, researchers found that the NLR, with a threshold of 3.5, emerged as a noteworthy prognostic biomarker for cancer patients with cachexia [20].

Nevertheless, the effects of including or excluding muscle mass in the definition remain uncertain, and there is insufficient data concerning the performance measures of alternative approaches in comparison to the original Fearon criteria. Our hypothesis is that the presence of cachexia, as defined by the Fearon criteria and these six approaches (omission of RMM, or utilizing alternative measures [MAMA, FFMI, CC, HGS and NLR]), will have the ability to predict mortality in individuals with cancer. Furthermore, these six modified criteria may demonstrate favourable performance and prove to be more feasible in the clinical environment. The objective of this study was to explore the associations between cachexia defined by different diagnostic criteria and the risk of mortality in a large population of patients with cancer. Additionally, we evaluated the frequency, indicators of diagnostic performance and concordance of the six modified criteria compared to the initial Fearon criteria. Finally, we investigated which diagnostic criteria were more feasible and appropriate for diagnosing cancer‐associated cachexia in clinical practice.

## 2 Methods

This present study is adhered to the Strengthening the Reporting of Observational Studies in Epidemiology (STROBE) Statement for cohort studies [21].

### 2.1 Study Design and Population

**FIGURE 1:**
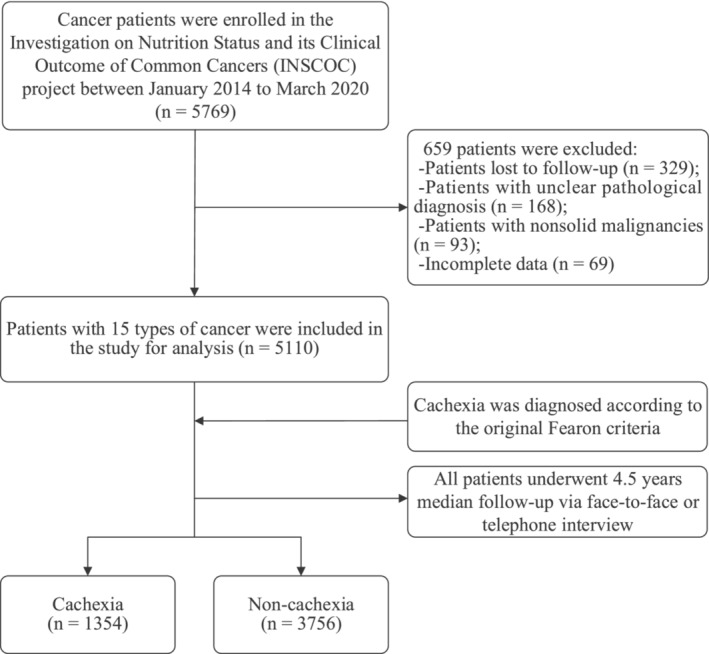
Flowchart diagram of the study design.

This was a multicentre, hospital‐based, prospective cohort study in China. Participants were obtained from a countrywide project, the Investigation on Nutrition Status and its Clinical Outcome of Common Cancers (INSCOC), which was registered on the website: http://www.chictr.org.cn/showproj.aspx?proj=31813 (identifier: ChiCTR1800020329). Previously, the complete INSCOC protocol has been published [22, 23], and the detailed inclusion and exclusion criteria can be found in Table S1. We selected a total of 5769 individuals who were at least 18 years old and had been diagnosed with cancer and/or were hospitalized for cancer in 72 tertiary hospitals across China between January 2014 and March 2020, based on the specified criteria. Every patient had comprehensive data on their weight loss for past 6 months, which was sufficient to diagnose cancer cachexia retrospectively. Patients with incomplete data (*n* = 69), nonsolid malignancies (*n* = 93), an unclear pathological diagnosis (*n* = 168) and loss to follow‐up (*n* = 329) were excluded additionally. Subsequently, we included a total of 5110 individuals diagnosed with 15 different types of cancer in our final analysis (Figure 1). A project‐trained technician conducted yearly follow‐up to gather survival data from each patient after enrolment through in‐person or phone interviews. The median follow‐up duration was 4.5 years. The most recent documented communication for follow‐up took place in October 2020. Approval for the study was granted by the Ethics Committees of all the institutions involved, and all patients provided written consent. The analysis of all data was conducted in an anonymous manner, ensuring the removal of any identifiable information, and adhering to the principles outlined in the Declaration of Helsinki.

### 2.2 Data Acquisition

Within 48 h of being admitted to the hospital, one or two proficient researchers at each institution obtained the fundamental data for all cancer patients, which was later extracted from the INSCOC database for analysis. We collected the following data at the time of admission: age, sex, family history of cancer, smoking status, alcohol consumption, clinical stage, involuntary weight loss in the past 6 month, Nutritional Risk Screening 2002 (NRS 2002) score [24], Patient‐Generated Subjective Global Assessment (PG‐SGA) score [25], European Organization for Research and Treatment of Cancer QLQ‐C30 (QLQ‐C30) score [26], laboratory test results using fasting blood samples (haemoglobin [HB], albumin [ALB], total protein [TP], prealbumin [PA], transferrin, total lymphocyte count [TLC], neutrophil count [NEU], white blood cell count [WBC] and NLR) and anthropometric measurements (height, weight, BMI, FFMI, ASMI, mid‐upper‐arm circumference [MAC], triceps skinfold thickness [TSF], MAMA, mid‐upper‐arm muscle circumference [MAMC], HGS and CC).

Inquiries were used to gather data on smoking status and alcohol consumption. Individuals who had consumed a minimum of 100 cigarettes throughout their lifetime, regardless of their current smoking status during the investigation, were classified as smokers. Alcohol consumption was defined as drinking alcohol at a frequency of at least once per week for a duration of 6 months. To evaluate unintentional weight loss, the patients were asked about their body weight 6 months prior and then this value was compared to the weight recorded upon hospital admission. Table S2 displays the comprehensive techniques and instruments employed for acquiring the anthropometric data (height, weight, BMI, FFMI, ASMI, MAC, TSF, MAMA, MAMC, HGS and CC). The electronic medical records were used to retrieve the clinical features documented during the hospital stay retrospectively, which encompassed the family history of cancer, clinical stage and the serum indices. All serum indices were measured by the participating institutions in their clinical laboratories using fasting blood samples. The TNM staging system was used to define pathological stages, and the classification adhered to the eighth edition of the American Joint Committee on Cancer (AJCC) cancer staging manual [27]. Each patient underwent a comprehensive interview with a dietitian or clinician to gather recent preoperative nutritional information, which included the NRS 2002 score, PG‐SGA score and the QLQ‐C30 score. Survival information was obtained through annual follow‐up assessments.

### 2.3 Definitions of Cancer Cachexia

#### 2.3.1 Fearon Criteria (Gold Standard)

According to Fearon et al.'s 2011 international consensus [6], cancer cachexia was diagnosed retrospectively if the patient fulfilled one or more of the following criteria: (1) involuntary weight loss more than 5% in the past 6 months, (2) any level of weight loss exceeding 2% and BMI below 18.5 kg/m^2^ (according to Asian standard for a low BMI) [28] and (3) any level of weight loss exceeding 2% and RMM (ASMI, males < 7.0 kg/m^2^ or females < 5.4 kg/m^2^, based on the AWGS 2019 Consensus) [16].

#### 2.3.2 Fearon Criteria With MAMA

Substituting the assessment of RMM in the Fearon criteria with MAMA, and the adjusted description of the third criterion is as follows: any level of weight loss exceeding 2% and a low MAMA (males < 32 cm^2^ or females < 18 cm^2^) [6].

#### 2.3.3 Fearon Criteria With FFMI

Substituting the assessment of RMM with FFMI, and the adjusted description of the third criterion is any level of weight loss exceeding 2% and a low FFMI (males < 14.6 kg/m^2^ or females < 11.4 kg/m^2^) [6].

#### 2.3.4 Fearon Criteria With CC

Substituting the assessment of RMM with CC, and the adjusted description of the third criterion is any level of weight loss exceeding 2% and a low CC (males < 30.5 cm or females < 29 cm) [15, 29].

#### 2.3.5 Fearon Criteria With HGS

Substituting the assessment of RMM with the measurement of muscle strength (HGS), and the adjusted description of the third criterion is any level of weight loss exceeding 2% and a low HGS (males < 22 kg or females < 16.1 kg) [17].

#### 2.3.6 Fearon Criteria With NLR

Substituting the assessment of RMM with the measurement of systemic inflammation (NLR), and the adjusted description of the third criterion is any level of weight loss exceeding 2% and a NLR ≥ 3.5 [20].

#### 2.3.7 Fearon Criteria Without RMM

Omission of the RMM as the third criterion within the definition of cancer cachexia, resulting in the consideration of only the first two criteria (weight loss > 5% or weight loss >2% + a low BMI) for the diagnosis.

#### 2.3.8 Statistical Analysis

To assess the normality of variable distributions, the Shapiro–Wilk test was employed. Quantitative variables were represented by the median and interquartile range (IQR) when abnormal distributions were observed, while qualitative variables were represented by the number of patients (percentage, %). The characteristics of all individuals were assessed at baseline. The total numbers of individuals with cancer cachexia diagnosed based on the seven different definitions were measured. To compare the continuous variables associated with the diagnosis of cancer cachexia (ASMI, MAMA, FFMI, CC, HGS, NLR and weight loss), a nonparametric Wilcoxon's rank‐sum test was employed, with sex being included as a stratification factor.

The survival was evaluated through the Kaplan–Meier analysis, and any disparities in survival were assessed using a stratified log‐rank test. Hazard ratios (HR) were used to assess the associations between cancer cachexia diagnosed using various definitions and survival. These associations were determined by employing multivariable‐adjusted Cox proportional hazard models, with 95% confidence intervals (CIs) included. Age, gender, clinical stage and cancer site were included as covariates for adjustment in the multivariable Cox analysis, because they are well‐established confounders.

The diagnostic properties of the six modified criteria were assessed using Fearon criteria as the reference standard. Performance indicators such as sensitivity, specificity, positive likelihood ratio, negative likelihood ratio, area under the ROC curve (AUC) and the Youden index were estimated. The AUC can range from 0.5 to 1.0 in numeric values, with a higher AUC indicating a greater level of accuracy in diagnostic performance [3].

Concordance between the Fearon criteria and the six modified criteria was assessed using the quadratic weighted kappa coefficients. Better agreement between the diagnostic tools is indicated by higher weighted kappa coefficients. The weighted kappa statistic ranges from 0.0 to 0.2, indicating slight consistency; 0.2 to 0.4, indicating fair consistency; 0.4 to 0.6, indicating moderate consistency; 0.6 to 0.8, indicating strong consistency; and 0.8 to 1.0, indicating almost perfect consistency [30, 31].

The significance of statistics was determined by considering *p* values less than 0.05 (two sided). The analyses were conducted utilizing SPSS (Version 26, IBM Corp, USA) and R (Version 3.6.3, http://www.rproject.org/).

## 3 Results

### 3.1 Baseline Characteristics of the Population

**FIGURE 2:**
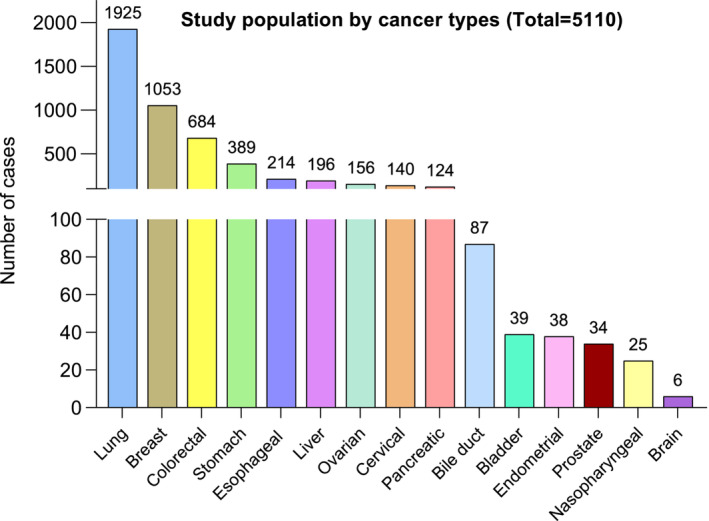
The study population stratified by the site of the cancer (*n* = 5110).

The overall column in Table 1 presents the complete baseline characteristics of the study cohort. After excluding ineligible individuals, this cohort included a total of 5110 patients, with a median age of 56 years. There were 2490 (48.7%) males and 2620 (51.3%) females. In 2237 (43.8%) patients, there was a record of tobacco use, and in 1014 (19.8%) patients, a record of alcohol consumption was noted. A total of 868 (17.0%) of the 5110 patients had a family history of cancer. Figure 2 shows that lung cancer was the most common diagnosis (37.7%) followed by cancers of the breast (20.6%), colorectum (13.4%), stomach (7.6%), oesophagus (4.2%) and liver (3.8%). Stage III (24.4%) and Stage IV (26.0%) were the most prevalent clinical stages. At baseline, a total of 1354 individuals (26.5%) met the Fearon criteria for cancer cachexia. Besides, the prevalence of cancer cachexia varied from 1115 (21.8%) individuals based on the Fearon criteria without RMM, to 1646 (32.2%) individuals based on the Fearon criteria with NLR when applying different definitions.

**TABLE 1:** Baseline characteristics of the study population stratified based on cachexia diagnosed by seven different approaches.

Characteristics	Total	Cachexia by Fearon criteria	Cachexia by Fearon criteria with MAMA	Cachexia by Fearon criteria with FFMI	Cachexia by Fearon criteria with CC	Cachexia by Fearon criteria with HGS	Cachexia by Fearon criteria with NLR	Cachexia by Fearon criteria without RMM
	*N* = 5110 (100%)	*N* = 1354 (26.5%)	*N* = 1338 (26.2%)	*N* = 1226 (24.0%)	*N* = 1367 (26.8%)	*N* = 1508 (29.5%)	*N* = 1646 (32.2%)	*N* = 1115 (21.8%)
Sex, *n* (%)								
Male	2490 (48.7%)	811 (59.9%)	829 (62.0%)	726 (59.2%)	785 (57.4%)	793 (52.6%)	937 (56.9%)	632 (56.7%)
Female	2620 (51.3%)	543 (40.1%)	509 (38.0%)	500 (40.8%)	582 (42.6%)	715 (47.4%)	709 (43.1%)	483 (43.3%)
Age, years, median (IQR)	56.0 (49.0–64.0)	57.0 (50.0–65.0)	57.0 (49.2–65.0)	57.0 (49.0–65.0)	58.0 (49.0–65.0)	58.0 (49.0–65.0)	57.5 (49.2–65.0)	57.0 (49.0–64.0)
Cancer family history, *n* (%)								
Yes	868 (17.0%)	215 (15.9%)	209 (15.6%)	189 (15.4%)	209 (15.3%)	230 (15.3%)	256 (15.6%)	178 (16.0%)
No	4242 (83.0%)	1139 (84.1%)	1129 (84.4%)	1037 (84.6%)	1158 (84.7%)	1278 (84.7%)	1390 (84.4%)	937 (84.0%)
Smoke, *n* (%)								
Yes	2237 (43.8%)	695 (51.3%)	715 (53.4%)	629 (51.3%)	695 (50.8%)	738 (48.9%)	809 (49.1%)	559 (50.1%)
No	2873 (56.2%)	659 (48.7%)	623 (46.6%)	597 (48.7%)	672 (49.2%)	770 (51.1%)	837 (50.9%)	556 (49.9%)
Alcohol consumption, *n* (%)								
Yes	1014 (19.8%)	337 (24.9%)	348 (26.0%)	307 (25.0%)	327 (23.9%)	330 (21.9%)	390 (23.7%)	273 (24.5%)
No	4096 (80.2%)	1017 (75.1%)	990 (74.0%)	919 (75.0%)	1040 (76.1%)	1178 (78.1%)	1256 (76.3%)	842 (75.5%)
Clinical stage, *n* (%)								
I	645 (12.6%)	142 (10.5%)	147 (11.0%)	127 (10.4%)	138 (10.1%)	164 (10.9%)	160 (9.72%)	122 (10.9%)
II	901 (17.6%)	209 (15.4%)	209 (15.6%)	196 (16.0%)	218 (15.9%)	242 (16.0%)	249 (15.1%)	181 (16.2%)
III	1249 (24.4%)	341 (25.2%)	343 (25.6%)	313 (25.5%)	355 (26.0%)	373 (24.7%)	431 (26.2%)	282 (25.3%)
IV	1327 (26.0%)	372 (27.5%)	358 (26.8%)	322 (26.3%)	360 (26.3%)	418 (27.7%)	485 (29.5%)	288 (25.8%)
NA	988 (19.3%)	290 (21.4%)	281 (21.0%)	268 (21.9%)	296 (21.7%)	311 (20.6%)	321 (19.5%)	242 (21.7%)
NRS 2002, median (IQR)	1 (0–1)	1 (0–3)	1 (0–3)	1 (0–4)	1 (0–3)	1 (0–3)	1 (0–3)	1 (0–4)
PG‐SGA, median (IQR)	4 (2–7)	6 (3–10)	6 (3–10)	6 (3–10)	6 (3–10)	6 (3–9)	5 (3–9)	7 (4–10)
Haematological measurements								
TP, g/L, median (IQR)	67.3 (62.5–71.7)	66.8 (62.3–71.5)	66.8 (62.3–71.3)	66.8 (62.3–71.3)	66.7 (62.0–71.0)	66.9 (62.3–71.4)	66.1 (61.0–70.8)	67.0 (62.4–71.4)
ALB, g/L, median (IQR)	39.0 (35.5–42.2)	38.8 (35.1–41.8)	38.6 (35.1–41.8)	38.6 (35.0–41.7)	38.4 (34.6–41.5)	38.6 (34.9–41.8)	37.7 (33.8–41.3)	39.0 (35.2–41.9)
PA, mg/L, median (IQR)	0.21 (0.17–0.25)	0.21 (0.17–0.25)	0.22 (0.17–0.26)	0.21 (0.17–0.25)	0.21 (0.16–0.25)	0.21 (0.17–0.25)	0.21 (0.16–0.25)	0.22 (0.17–0.26)
HB, g/L, median (IQR)	125 (110–138)	126 (110–139)	127 (110–139)	126 (109–138)	125 (108–138)	125 (108–138)	123 (106–137)	127 (111–139)
Transferrin, mg/L, median (IQR)	2.20 (1.84–2.60)	2.16 (1.79–2.57)	2.16 (1.80–2.57)	2.15 (1.79–2.57)	2.15 (1.78–2.56)	2.17 (1.79–2.58)	2.09 (1.74–2.52)	2.16 (1.81–2.57)
WBC, × 10^9^/L, median (IQR)	5.90 (4.46–7.75)	5.97 (4.42–7.96)	5.96 (4.46–7.94)	5.94 (4.42–7.92)	6.04 (4.48–8.07)	6.02 (4.46–8.02)	6.54 (4.82–8.80)	5.93 (4.42–7.86)
NEU, × 10^9^/L, median (IQR)	3.54 (2.35–5.17)	3.70 (2.35–5.41)	3.68 (2.36–5.35)	3.67 (2.35–5.34)	3.78 (2.36–5.56)	3.69 (2.36–5.40)	4.34 (2.72–6.50)	3.62 (2.34–5.27)
TLC, × 10^9^/L, median (IQR)	1.50 (1.07–1.97)	1.47 (1.06–1.95)	1.46 (1.06–1.93)	1.46 (1.05–1.93)	1.44 (1.03–1.93)	1.44 (1.06–1.93)	1.32 (0.91–1.76)	1.47 (1.06–1.94)
NLR, median (IQR)	2.33 (1.52–3.80)	2.43 (1.59–3.86)	2.45 (1.58–3.98)	2.48 (1.59–3.99)	2.54 (1.61–4.11)	2.45 (1.58–3.99)	3.61 (1.94–5.60)	2.41 (1.58–3.86)
Anthropometric measurements								
Height, cm, median (IQR)	165 (158–170)	166 (160–171)	166 (160–171)	166 (160–171)	165 (159–170)	165 (159–170)	165 (160–171)	165 (160–170)
Weight, kg, median (IQR)	62.0 (55.0–70.0)	62.0 (54.5–69.5)	62.0 (55.0–70.0)	61.3 (53.7–69.5)	60.0 (52.5–68.0)	60.0 (52.0–67.7)	61.6 (54.0–69.6)	62.0 (55.0–70.0)
BMI, kg/m^2^, median (IQR)	23.1 (20.7–25.4)	22.6 (20.2–24.9)	22.6 (20.3–25.0)	22.4 (19.8–25.0)	22.0 (19.5–24.7)	22.0 (19.7–24.7)	22.5 (20.1–25.0)	22.8 (20.4–25.2)
FFMI, kg/m^2^, median (IQR)	15.8 (14.6–17.2)	15.9 (14.7–17.2)	16.0 (14.8–17.3)	15.8 (14.4–17.2)	15.7 (14.4–17.0)	15.6 (14.3–16.9)	15.8 (14.6–17.2)	16.0 (14.8–17.3)
ASMI, kg/m^2^, median (IQR)	7.01 (6.30–7.70)	7.17 (6.42–7.73)	7.24 (6.46–7.76)	7.11 (6.42–7.70)	7.02 (6.29–7.65)	6.94 (6.14–7.61)	7.12 (6.34–7.72)	7.17 (6.39–7.76)
MAC, cm, median (IQR)	26.8 (24.5–28.8)	26.0 (24.0–28.5)	26.3 (24.4–28.5)	26.0 (24.0–28.5)	26.0 (23.6–28.0)	26.0 (24.0–28.0)	26.0 (24.0–28.4)	26.5 (24.5–28.5)
TSF, mm, median (IQR)	18.0 (12.0–22.0)	16.0 (12.0–22.0)	16.0 (12.0–22.0)	16.0 (12.0–22.0)	16.0 (11.4–21.0)	16.0 (12.0–21.0)	16.0 (12.0–22.0)	16.0 (12.0–22.0)
MAMC, cm, median (IQR)	21.1 (19.3–23.0)	21.1 (19.2–23.0)	21.1 (19.3–23.0)	21.1 (19.2–23.0)	20.8 (18.9–22.8)	20.7 (19.0–22.7)	21.0 (19.2–22.9)	21.2 (19.3–23.0)
MAMA, cm^2^, median (IQR)	35.4 (29.8–42.0)	35.4 (29.5–42.0)	35.5 (29.6–42.1)	35.3 (29.2–41.9)	34.5 (28.5–41.5)	34.2 (28.6–41.1)	35.0 (29.4–41.6)	35.8 (29.7–42.2)
CC, cm, median (IQR)	34.0 (31.3–36.2)	33.3 (30.8–36.0)	33.5 (31.0–36.0)	33.5 (30.8–36.0)	33.0 (29.6–35.5)	32.0 (30.0–35.0)	33.4 (31.0–36.0)	34.0 (31.0–36.0)
HGS, kg, median (IQR)	23.3 (17.6–30.5)	24.5 (18.5–31.5)	24.7 (18.8–31.7)	24.2 (18.3–31.2)	23.5 (17.8–30.7)	23.0 (17.1–30.1)	23.6 (17.6–31.0)	24.5 (18.7–31.6)
Weight loss, *n* (%)								
Yes	2547 (49.8%)	693 (51.2%)	680 (50.8%)	668 (54.5%)	809 (59.2%)	775 (51.4%)	1088 (66.1%)	557 (50.0%)
No	2563 (50.2%)	661 (48.8%)	658 (49.2%)	558 (45.5%)	558 (40.8%)	733 (48.6%)	558 (33.9%)	558 (50.0%)
QOL by QLQ‐C30, median (IQR)	38.5 (35.9–41.9)	38.6 (35.7–42.3)	38.5 (35.6–42.1)	38.5 (35.6–42.3)	38.5 (35.9–42.3)	38.5 (35.6–42.3)	38.5 (35.9–42.3)	38.6 (35.6–42.3)

*Note:* Data are represented as the median (interquartile range) or number (%).

Abbreviations: ALB, albumin; ASMI, appendicular skeletal muscle index; BMI, body mass index; CC, calf circumference; FFMI, fat‐free mass index; HB, haemoglobin; HGS, hand grip strength; IQR, interquartile range; MAC, mid‐upper‐arm circumference; MAMA, mid‐upper‐arm muscle area; MAMC, mid‐upper‐arm muscle circumference; NA, not applicable; NEU, neutrophil count; NLR, neutrophil‐to‐lymphocyte ratio; NRS 2002, Nutritional Risk Screening 2002; PG‐SGA, Patient‐Generated Subjective Global Assessment; PA, prealbumin; QOL, quality of life; QLQ‐C30, the European Organization for Research and Treatment of Cancer (EORTC) QLQ‐C30 score; RMM, reduced muscle mass; TLC, total lymphocyte count; TP, total protein; TSF, triceps skinfold thickness; WBC, white blood cell count.

**FIGURE 3:**
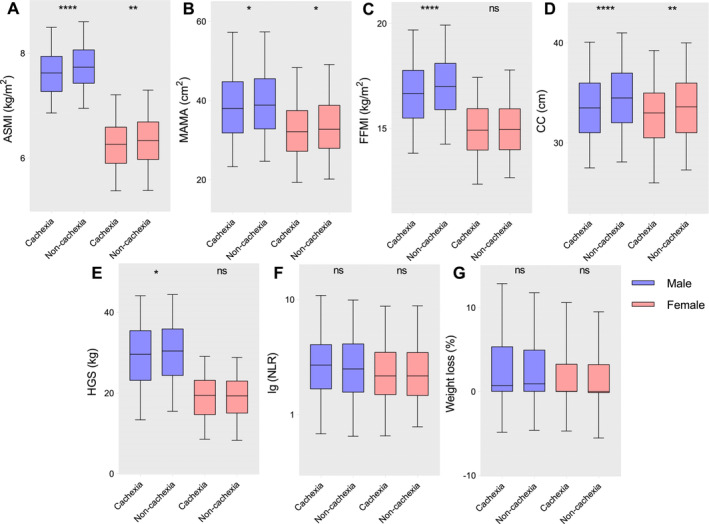
The distribution of indicators related to muscle mass and systemic inflammation in patients with different disease status (cachexia vs. noncachexia) stratified based on sex (male vs. female). (A) ASMI. (B) MAMA. (C) FFMI. (D) CC. (E) HGS. (F) NLR. (G) Weight loss.

We analysed the distribution of variables related to muscle mass and systemic inflammation in patients with different disease status (cachexia vs. noncachexia) and different sex (male vs. female). It was found that in the cachexia group defined by original Fearon criteria, the ASMI, MAMA, FFMI, CC and HGS of male patients were lower than those in noncachexia group significantly (*p* < 0.0001, *p* < 0.05, *p* < 0.0001, *p* < 0.0001 and *p* < 0.05, respectively). In Figure 3, the ASMI, MAMA and CC of female patients in cachexia group defined by original Fearon criteria were also lower than those in noncachexia group significantly (*p* < 0.01, *p* < 0.05 and *p* < 0.01, respectively).

### 3.2 Kaplan–Meier Analysis

**FIGURE 4:**
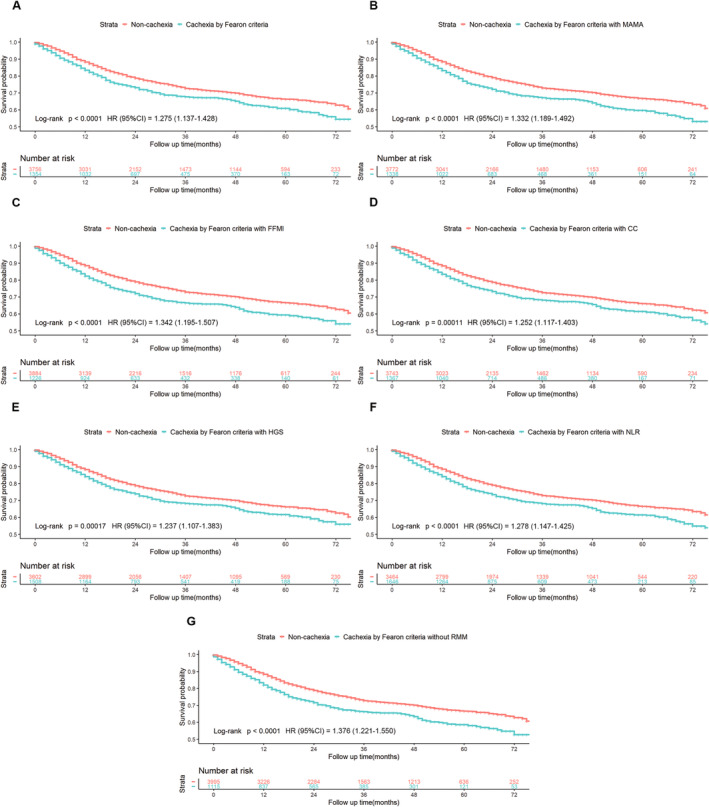
Kaplan–Meier curves for cancer patients with/without cachexia diagnosed based on the seven different definitions. (A) The original Fearon criteria. (B) Fearon criteria with MAMA. (C) Fearon criteria with FFMI. (D) Fearon criteria with CC. (E) Fearon criteria with HGS. (F) Fearon criteria with NLR. (G) Fearon criteria without RMM.

In this study, the 5110 individuals were with 4.5 years median follow‐up duration, and 1412 (27.6%) died during this period. The Kaplan–Meier curves in Figure 4 display the outcomes of cancer patients with and without cachexia, as determined by seven different definitions. All of the seven definitions for cancer cachexia can clearly separate the survival curves for cachexia/noncachexia patients. The crude HR varied from the minimum HR acquired using Fearon criteria with HGS (HR, 1.237; 95% CI, 1.107–1.383; log‐rank *p*, 0.00017) to the maximum acquired using Fearon criteria without RMM (HR, 1.376; 95% CI, 1.221–1.550; log‐rank *p* < 0.0001).

### 3.3 Multivariable Survival Analysis

**FIGURE 5:**
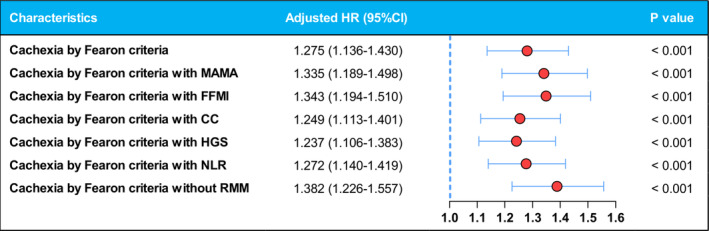
The association between cancer cachexia diagnosed by different definitions and survival. Adjusted by age, sex, clinical stage and cancer site.

Figure 5 displays the results of the multivariable‐adjusted Cox proportional hazards model analyses, regarding associations between survival and cancer cachexia diagnosed using various definitions. After adjustment for age, sex, clinical stage and cancer site, individuals who fulfilled the original Fearon criteria exhibited a significant increase in the risk of mortality (HR, 1.275; 95% CI, 1.136–1.430; *p* < 0.001). In the adjusted model, individuals who fulfilled any of the six modified Fearon criteria also exhibited a significant increase in the risk of mortality. The adjusted HR varied from the lowest HR acquired by Fearon criteria with HGS (HR, 1.237; 95% CI, 1.106–1.383; *p* < 0.001) to the highest acquired by Fearon criteria without RMM (HR, 1.382; 95% CI, 1.226–1.557; *p* < 0.001).

### 3.4 Diagnostic Performance of Different Criteria for Cachexia

Table 2 displays the performance indicators of the six modified Fearon criteria in comparison to the original Fearon criteria, considered as the gold standard. All of the six modified Fearon criteria had a sensitivity and a specificity > 80%. The lowest sensitivity was acquired using the Fearon criteria without RMM (82.4%; 95% CI, 80.2%–84.3%) and the highest by the Fearon criteria with HGS (90.7%; 95% CI, 89.0%–92.2%). The lowest specificity was obtained by the Fearon criteria with NLR (86.9%; 95% CI, 85.7%–87.9%) and the highest by the Fearon criteria without RMM (100.0%; 95% CI, 99.9%–100.0%).

**TABLE 2:** Performance indicators of different approaches of cachexia diagnostic criteria compared with Fearon criteria (gold standard).

	Sensitivity (95% CI, %)	Specificity (95% CI, %)	Positive likelihood ratio (95% CI)	Negative likelihood ratio (95% CI)	AUC (95% CI)	Youden index	*p*
Fearon criteria with MAMA	89.7 (88.0–91.3)	96.7 (96.1–97.3)	27.402 (23.009–32.634)	0.106 (0.091–0.124)	0.932 (0.925–0.939)	0.865	< 0.001
Fearon criteria with FFMI	83.8 (81.7–85.7)	97.6 (97.0–98.0)	34.193 (27.906–41.896)	0.167 (0.148–0.188)	0.907 (0.898–0.914)	0.813	< 0.001
Fearon criteria with CC	85.2 (83.2–87.1)	94.3 (93.5–95.0)	15.029 (13.167–17.155)	0.157 (0.138–0.178)	0.898 (0.889–0.906)	0.796	< 0.001
Fearon criteria with HGS	90.7 (89.0–92.2)	92.6 (91.7–93.4)	12.166 (10.856–13.635)	0.101 (0.085–0.119)	0.916 (0.908–0.924)	0.832	< 0.001
Fearon criteria with NLR	85.1 (83.1–86.9)	86.9 (85.7–87.9)	6.469 (5.941–7.044)	0.172 (0.151–0.195)	0.860 (0.850–0.869)	0.719	< 0.001
Fearon criteria without RMM	82.4 (80.2–84.3)	100.0 (99.9–100.0)	—	0.177 (0.157–0.198)	0.912 (0.904–0.919)	0.824	< 0.001

Abbreviations: AUC, area under the curve; CC, calf circumference; FFMI, fat‐free mass index; HGS, hand grip strength; MAMA, mid‐upper‐arm muscle area; NLR, neutrophil‐to‐lymphocyte ratio; RMM, reduced muscle mass.

The positive likelihood ratio of the six modified criteria ranged from 6.469 (95% CI, 5.941–7.044) obtained by the Fearon criteria with NLR to 34.193 (95% CI, 27.906–41.896) obtained by the Fearon criteria with FFMI. The negative likelihood ratio ranged from 0.101 (95% CI, 0.085–0.119) to 0.177 (95% CI, 0.157–0.198).

All the AUCs of the six modified Fearon criteria were > 0.8, ranging from 0.860 (95% CI, 0.850–0.869) obtained by the Fearon criteria with NLR to 0.932 (95% CI, 0.925–0.939) obtained by the Fearon criteria with MAMA. The Youden index of the six modified criteria ranged from 0.719 obtained by the Fearon criteria with NLR to 0.865 obtained by the Fearon criteria with MAMA. When adopting the Fearon criteria without RMM for the diagnosis of cachexia, the AUC and Youden index were 0.912 (95% CI, 0.904–0.919) and 0.824, respectively.

### 3.5 Concordance Analysis

**FIGURE 6:**
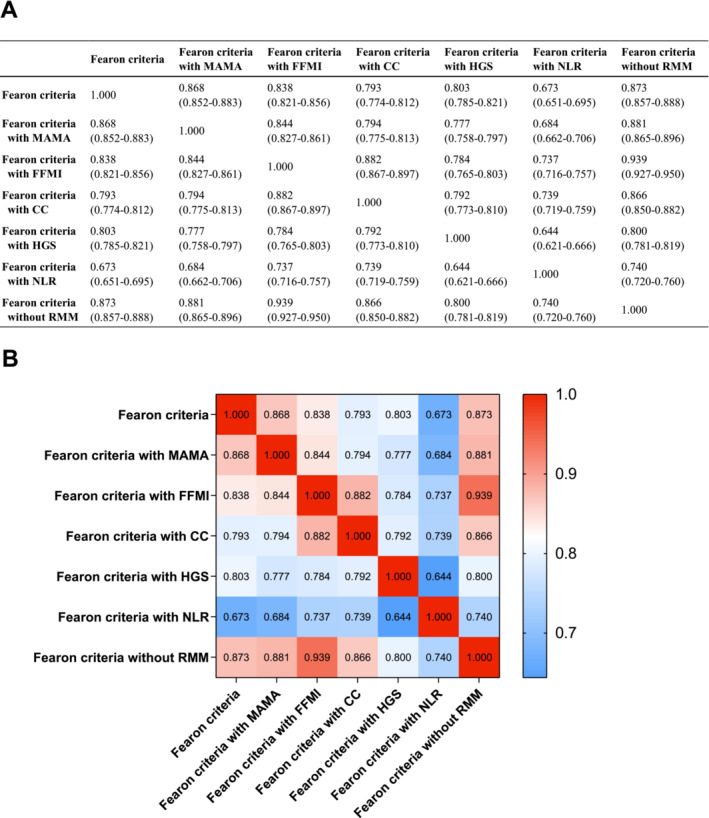
(A) Kappa concordance analysis between the original Fearon criteria and the six pragmatic modified criteria. (B) Heatmap of the concordance analysis.

The concordance between the Fearon criteria and six modified criteria was evaluated by calculating the quadratic weighted kappa coefficients, and the results among all of them are shown in Figure 6. The values of the quadratic kappa coefficient ranged from 0.673 (95% CI, 0.651–0.695, strong) between the Fearon criteria and the Fearon criteria with the NLR to 0.873 (95% CI, 0.857–0.888, almost perfect) between the Fearon criteria and the Fearon criteria without RMM (Figure 6A). We also conducted a heatmap to present the results of concordance analysis in a visualized method (Figure 6B).

To further eliminate the confounding impact of the first two criteria (weight loss > 5% and a low BMI + weight loss >2%) on the concordance, we conducted an additional concordance analysis after excluding the 1115 cachexia patients identified based on solely the weight loss > 5% or low BMI + weight loss >2% criteria. The quadratic weighted kappa coefficients for this analysis are shown in Figure S1. Unsurprisingly, all of the kappa coefficients were decreased, but fair consistency remained between the original Fearon criteria and the Fearon criteria with MAMC or HGS.

## 4 Discussion

This was an extensive, multicentre, hospital‐based, prospective cohort study that included 5769 participants with 15 types of cancers across China. This study focused on a real‐world clinical challenge: It is hard for clinicians to diagnose cancer cachexia without large expensive instruments, especially in developing countries. This is the first large‐scale investigation that addressed this challenge and systematically explored a simple feasible means to replace ASMI in the muscle mass assessment for diagnosing cancer cachexia. Our present findings could potentially assist healthcare professionals, including clinicians or nutritionists, in making informed decisions regarding the treatment of cancer cachexia. Additionally, these findings may offer valuable insights for developing effective management strategies to enhance clinical outcomes.

The diagnosis of cancer cachexia commonly involves identifying unintentional weight loss, a low BMI and skeletal muscle exhaustion, which are often associated with systemic inflammation and reduced intake [6, 32]. Research by Vaughan et al. revealed that 86% of cancer patients experience cachexia in the final 1–2 weeks of their lives, with about half of them experiencing a weight loss of over 10% throughout the disease [33]. Several studies have indicated that inflammation not only contributes to tumour growth and metastasis but also plays a noteworthy role in the energy imbalance and muscle wasting associated with cancer cachexia [20, 34, 35]. A previous cohort study that included 2470 participants with nonmetastatic colorectal cancer demonstrated that systemic inflammation was linked to muscle exhaustion, and the group with an elevated NLR had worse progression‐free and overall survival rates [36]. Hence, in this study, we also discussed the possibility of using the NLR as an indicator of systemic inflammation to replace RMM in diagnosing of cancer cachexia. In the Fearon's framework, we also evaluated the effectiveness of utilizing the HGS, a muscle strength indicator, as a substitute for muscle mass. The HGS is a straightforward and nonintrusive anthropometric measurement of muscle strength, and numerous studies have suggested that a low HGS is a significant marker for defining sarcopenia and cancer cachexia in clinical settings [37, 38].

Generally speaking, the Fearon's framework is a consensus by the Delphi method raised more than 10 years ago. However, due to the ever‐changing nature of the field, there is currently no universally agreed upon set of diagnostic criteria for cancer cachexia. A main challenge the scientific community faces is to optimize and identify effective diagnostic criteria from a multitude of possible contenders. There are three reasons why our study could have a hastening effect on the update of the Fearon criteria. First, our study demonstrated that the Fearon criteria have the ability to forecast mortality in individuals with cancer, thus confirming the utility of the framework put forth by Fearon et al. in the original reference. In addition, all six modified criteria were also able to forecast mortality, with performance similar to the original Fearon framework. Second, our study provided acceptable new approaches for the replacement of measuring RMM, due to the absence of appropriate technical equipment in the majority of clinical environments [11]. Third, our study showed that even excluding muscle mass from the definition, weight loss and a low BMI remained reliable indicators of cancer cachexia‐related mortality. Recognition and reappraisal may be necessary for understanding the full significance of body weight and BMI in predicting the outcome of individuals suffering from cancer cachexia [37, 39].

During the current investigation, cancer cachexia was considered to be present in 21.8%–32.2% of patients according to different definitions. It is noteworthy that the incidence of cachexia obtained by Fearon criteria without RMM is 21.8%, lowest among all of the seven different criteria. Compared with the original Fearon criteria (26.5%), omission of the muscle mass in the definition might increase the risk of a missed diagnosis of cachexia. However, through multivariable Cox analysis, we found that not only the original Fearon criteria (HR, 1.275; 95% CI, 1.136–1.430; *p* < 0.001) but also the six modified criteria (HR, 1.237–1.382; *p* < 0.001) could predict the survival of cancer patients. In addition, when the muscle mass was omitted in the definition, the adjusted HR (1.382; 95% CI, 1.226–1.557; *p* < 0.001) was the highest among all the seven criteria. This finding indicated that Fearon criteria without RMM (a low BMI + weight loss >2% or weight loss >5%) outperformed the original Fearon criteria and other substitution in predicting cancer survival, at least among the present cohort representative of the Chinese population.

The indicators of diagnostic performance for the six different criteria compared with the original Fearon criteria are listed in Table 2. All of the modified Fearon criteria had a sensitivity, specificity and AUC > 80%, which demonstrated that all six of the modified criteria presented favourable diagnostic performance in distinguishing cancer cachexia. In the concordance analysis (Figure 6), all of the modified criteria showed strong to almost perfect consistency with the original Fearon criteria, with high kappa coefficients ranging from 0.673 (95% CI, 0.651–0.695) to 0.873 (95% CI, 0.857–0.888). After excluding 1115 cachexia patients who were identified based on the weight loss > 5% or low BMI + weight loss >2% criteria, an additional concordance analysis was performed. The results in Figure S1 demonstrate that there was fair concordance between the original Fearon criteria and the criteria with MAMA (*κ* = 0.398), the original Fearon criteria and the criteria with HGS (*κ* = 0.306), the criteria with FFMI and the criteria with CC (*κ* = 0.344), the criteria with CC and the criteria with HGS (*κ* = 0.276) and the criteria with CC and the criteria with NLR (*κ* = 0.222). This additional analysis reflects the connections and variability among different alternative indicators and emphasizes the importance of considering clinical context and resource availability.

The NLR appeared to exhibit lower accuracy and greater variability compared to other muscle measurements in the AUC analysis and concordance analysis. However, the NLR reflects the nonspecific immunity/inflammation, which can be elevated in cancer patients due to various factors like burns, trauma, major infections and closed head injury, potentially leading to a misdiagnosis of cachexia. Nevertheless, systemic inflammation is a well‐recognized component in the pathophysiology of cancer cachexia, contributing to muscle wasting and metabolic alterations [6, 20]. Despite the above‐referenced other causes of an elevated NLR, the accuracy and concordance of substituting RMM with NLR in diagnosing cachexia were still acceptable in our study. This indicates that NLR can be a practical alternative, especially in settings where measurements of the other features (muscle mass, CC, HGS, etc.) are not readily available due to a lack of training or insufficient personnel resources. This aligns with previous research suggesting that systemic inflammation markers, including the NLR, are associated with cachexia and may have prognostic value in cancer patients [20]. However, further studies are warranted to validate the efficacy of the NLR and other inflammatory markers in diverse clinical settings and to determine their role in comprehensive diagnostic frameworks for cancer cachexia.

The modified Fearon criteria with HGS had a slightly higher sensitivity in our study compared to other modified Fearon criteria. This might suggest that the HGS could capture subtle impairments in function that occur in the early stages of cancer cachexia. However, other performance indicators, including the AUC and HR, were comparable across the different criteria, indicating that their overall utilities in diagnosing cachexia and predicting survival are broadly similar. It is important to interpret these findings with caution, as the observed differences may not be clinically significant.

A previous study published in 2017 by Blauwhoff‐Buskermolen et al. [40], which included 241 patients with advanced cancers such as lung (36%), colon/rectum (31%), prostate (18%) and breast (15%) cancers, investigated the effects of different muscle mass measurement methods on the diagnosis of cancer cachexia. The researchers found that the type of muscle mass measurement significantly affected the prevalence of low muscle mass, but it had little influence on the overall diagnosis of cancer cachexia. This finding was largely attributed to the fact that most patients in their study (67.8%–88.6%) had already been identified as cachexia solely based on the criteria of weight loss > 5%.

In our examination of 1354 patients diagnosed with cachexia according to the original Fearon criteria, we also found that weight loss was a predominant factor, with 1040 (76.8%) of these patients meeting this criterion (weight loss > 5%). This finding underscores the robustness of weight loss‐based criteria in identifying cachexia. By expanding the sample size to 5110 participants from a Chinese multicentre cohort, our study not only confirms the findings reported by Blauwhoff‐Buskermolen et al. but also emphasizes the utility of alternative, accessible and less costly muscle mass measurements or inflammation markers in diagnosing cachexia, particularly in resource‐limited settings. In addition, in our population, completely eliminating the RMM from the Fearon criteria (keeping just the weight loss > 5% or weight loss > 2% + low BMI) still yielded relatively high sensitivity and specificity for cancer cachexia. The present findings suggest that although incorporating additional parameters (any of the indicators of muscle mass or inflammation) increases the number of identified cases, if these parameters are unavailable, a minimal assessment should still be performed using only the patient's reported weight loss and current BMI. This approach will still identify the majority of patients with cancer cachexia.

There are several potential limitations in the present study. First, the current investigation determined the weight reduction by utilizing the patient's self‐reported previous weight. Therefore, it is impossible to eliminate the influence of recall bias on the categorization of cancer cachexia when utilizing Fearon's framework. Second, it should be noted that the results of this observational cohort study, which included cancer patients from various regions of China, may primarily pertain to the conditions found in Chinese tertiary hospitals. The applicability of the results to other populations may vary. Third, the ASMI in the present study was acquired from validated anthropometric equation, instead of cross‐sectional imaging or DEXA. Nonetheless, previous evidence has shown that the anthropometric equation exhibits good fitness with DEXA [41]. To validate our findings, future studies should employ more sophisticated techniques for assessing muscle mass. Fourth, given the anthropometric disparities between Asians and Western populations [16], it is necessary to reassess the prognostic significance of anthropometric measurements when utilized in patients who are not of Asian descent or who observe other dietary patterns.

## 5 Conclusions

In conclusion, our study provided six modified methods based on the Fearon criteria for the diagnosis of cachexia, which predicted the survival of cancer patients, presented adequate performance indicators, had strong to almost perfect consistency with the original criteria and were feasible. In the clinical setting, clinicians and nutritionists would choose the diagnostic criteria available to them and/or easily obtained in their clinic.

## Ethics Statement

All authors certify that the manuscript conforms to the ethical guidelines for publishing in the *Journal of Cachexia, Sarcopenia and Muscle*. National and international research ethics guidelines were followed, including the Deontological Code of Ethics and the 1964 Declaration of Helsinki and its later amendments. All patients provide written consent for the use of their data, and this study was approved by the institutional Ethics Committee of all participating institutions.

## Conflicts of Interest

The authors declare no conflicts of interest.

## Supporting information


**Figure S1** Heatmap of concordance analysis between Fearon criteria and the five pragmatic modified criteria, excluding 1115 cancer cachexia patients diagnosed by the first two criteria (weight loss > 5% or BMI criteria + weight loss > 2%).

**Table S1** Inclusion and exclusion criteria for the INSCOC project.

**Table S2** Procedures and devices used to obtain the anthropometric parameters in the present study.

## Data Availability

Individual data will be made available following publication by reasonable request to the corresponding author. The study protocol of INSCOC was registered online at http://www.chictr.org.cn/showproj.aspx?proj=31813.
