# Supplementary material for: Diagnostic Criteria for Cancer‐Associated Cachexia: Insights from a Multicentre Cohort Study
Source: J Cachexia Sarcopenia Muscle. 2025 Feb 13;16(1):e13703. doi: 10.1002/jcsm.13703 (PMC11825978; doi:10.1002/jcsm.13703)

**Supplemental Figure 1.** Heatmap of concordance analysis between Fearon criteria and the 5 pragmatic modified criteria, excluding 1115 cancer cachexia patients diagnosed by the first two criteria (weight loss＞5% or BMI criteria + weight loss＞2%)


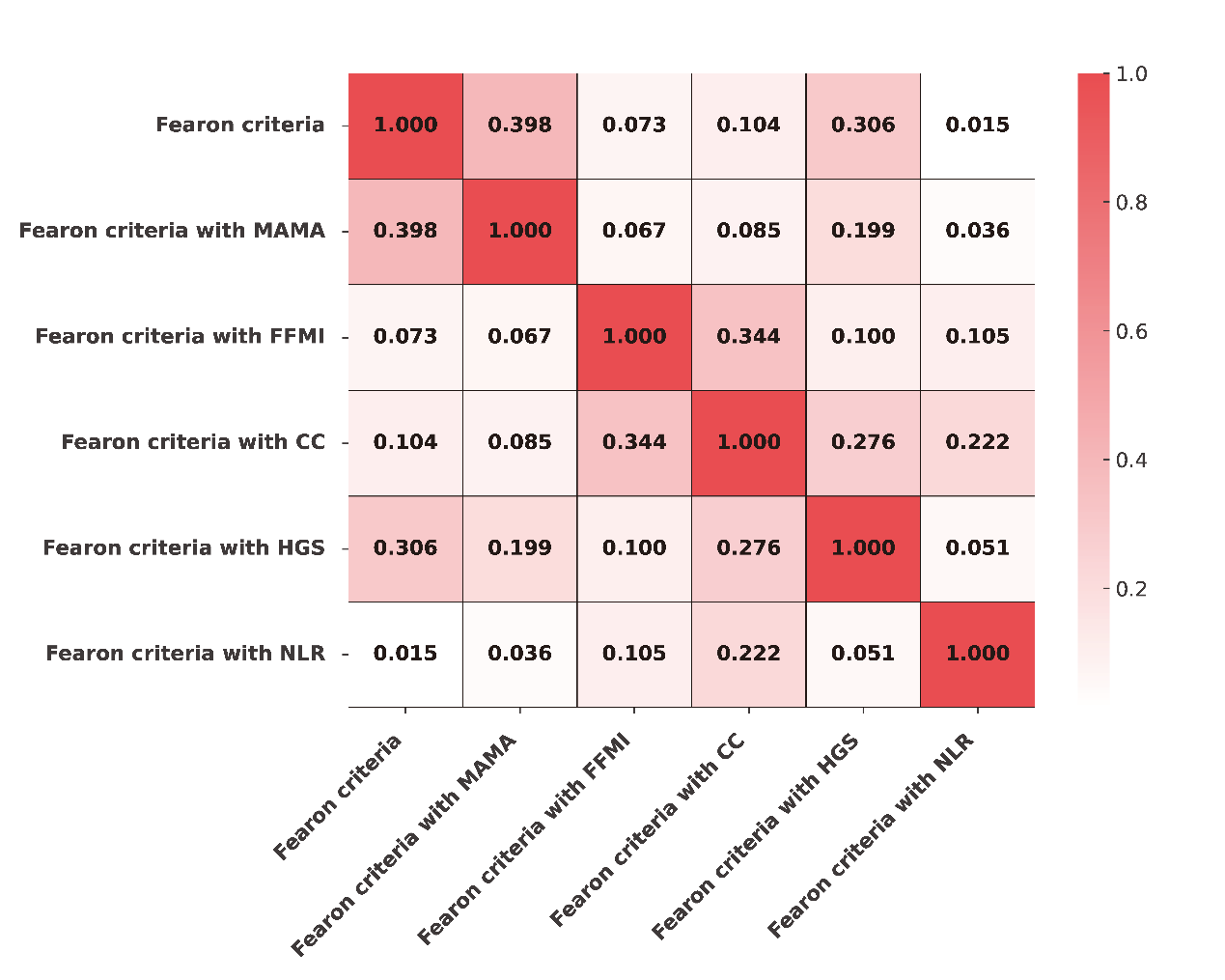

Supplement: Supplementary file 1 — Figure S1 Heatmap of concordance analysis between Fearon criteria and the five pragmatic modified criteria, excluding 1115 cancer cachexia patients diagnosed by the first two criteria (weight loss > 5% or BMI criteria + weight loss > 2%). [file JCSM-16-e13703-s003.docx]
