# Supplementary material for: Diagnostic Criteria for Cancer‐Associated Cachexia: Insights from a Multicentre Cohort Study
Source: J Cachexia Sarcopenia Muscle. 2025 Feb 13;16(1):e13703. doi: 10.1002/jcsm.13703 (PMC11825978; doi:10.1002/jcsm.13703)
Supplement: Supplementary file 2 — Table S1 Inclusion and exclusion criteria for the INSCOC project. [file JCSM-16-e13703-s001.docx]

**Supplemental Table 1.** Inclusion and exclusion criteria for the INSCOC project

| **Inclusion criteria** | **Exclusion criteria** |
| --- | --- |
| 1) Age at least 18 years;  2) With length of hospital stay longer than 48 hours;  3) Diagnosed with one of the following 18 types of locally or metastatic malignant tumors: lung cancer, gastric cancer, liver cancer, colorectal cancer, breast cancer, esophageal cancer, cervical cancer, endometrial cancer, nasopharyngeal carcinoma, malignant lymphoma, leukemia, pancreatic cancer, ovarian cancer, prostate cancer, bladder cancer, brain tumors, biliary tract malignant tumors and gastrointestinal stromal tumors. | 1) With organ transplantation;  2) Pregnant woman;  3) Diagnosed with HIV infection or AIDS;  4) Admitted to the ICU at the beginning of recruitment;  5) If patients were hospitalized more than two times during the investigation, only the data from the first survey were included. |

Abbreviations: INSCOC, Investigation on Nutrition Status and its Clinical Outcome of Common Cancers (chictr.org.cn: ChiCTR1800020329); HIV, human immunodeficiency virus; AIDS, acquired immunodeficiency syndrome; ICU, intensive care unit.
