# Supplementary material for: Diagnostic Criteria for Cancer‐Associated Cachexia: Insights from a Multicentre Cohort Study
Source: J Cachexia Sarcopenia Muscle. 2025 Feb 13;16(1):e13703. doi: 10.1002/jcsm.13703 (PMC11825978; doi:10.1002/jcsm.13703)
Supplement: Supplementary file 3 — Table S2 Procedures and devices used to obtain the anthropometric parameters in the present study. [file JCSM-16-e13703-s002.docx]

**Supplemental Table 2.** Procedures and devices used to obtain the anthropometric parameters in the present study

| **Parameter** | **Instrument** | **Method** |
| --- | --- | --- |
| Body weight six months before admission (kg) | Not applicable | Patient-reported during an in-person interview, within 48 hours of the first admission. |
| Body weight (kg) and height (cm) at baseline | Height & weight measuring instrument (Model SH-201, Zhengzhou, China) | The body weight and height of patients were measured using an integrated height and weight measuring instrument in patients wearing light indoor clothing without shoes, to the nearest 0.1 kg and 0.1 cm, respectively, within 48 hours of the first admission. |
| Weight loss | Not applicable | (𝑤𝑒𝑖𝑔ℎ𝑡_updated_ − 𝑤𝑒𝑖𝑔ℎ𝑡_previous_) / 𝑤𝑒𝑖𝑔ℎ𝑡_previous_ × 100% |
| Body mass index (kg/m^2^) | Not applicable | The body mass index was calculated as the weight in kilograms divided by the height in meters squared. |
| Mid-arm Circumference (MAC, non-dominant arm, cm) | A flexible and non-elastic tape | The MAC (non-dominant arm) was measured using a flexible and non-elastic tape to the nearest 0.1 cm. The MAC was measured while the patient was in a standing position with the non-dominant arm and the elbow relaxed. The measuring tape was placed around the upper arm at the midpoint perpendicular to the long axis of the upper arm and the value was recorded. When measuring the MAC, the tape was pressed to the skin surface without tight compression. |
| Triceps skinfold thickness (TSF, non-dominant arm, mm) | Adipometer (PZJ-01, Jiangsu, China) | The TSF was measured while the patient was in a seated position with shoulders relaxed and arms hanging freely at the sides. The researcher pinched about 2.0 cm of the triceps skinfold and measured it using the adipometer with its jaws held perpendicular to the shaft of the arm. |
| Mid-arm muscle area (MAMA) | Not applicable | MAMA (cm^2^) = [MAC (cm) - 3.14 × TSF (cm)] ^2 / (4 × 3.14) |
| Mid-arm muscle circumference (MAMC) | Not applicable | MAMC (cm) = MAC (cm) - 3.14 × TSF (cm) |
| Hand grip strength (HGS) | Handgrip Dynamometer (CAMRY, Model EH101, Guangdong, China) | Patients were asked to stand comfortably, then to perform three maximal isometric contractions 30s apart using their non-dominant hand. The maximum read for the HGS was recorded. |
| Calf circumference (CC, left calf, cm) | A flexible and non-elastic tape | The calf circumference (CC, left calf) was measured using a flexible and non-elastic tape to the nearest 0.1 cm. The CC was measured while the patient was in a seated position, with the measurement taken on a perpendicular plane to the long axis of the left calf to obtain the maximal value. When measuring the CC, the tape was pressed to the skin surface without tight compression. |
| Fat free mass index (FFMI) | Bioelectrical impedance device (Inbody 720 /Inbody S10, Seoul, Korea) | The FFMI was measured using a bioelectrical impedance body composition analyzer in patients wearing light indoor clothing without shoes. The recommended measurement posture for Inbody 720 is standing, whereas for patients who cannot maintain a standing posture, sitting or supine positions are permitted for S10. Each participant was assessed at least once. |
| Appendicular skeletal muscle mass (ASM, kg) | Not applicable | ASM was estimated according to a previously validated anthropometric equation: ASM = - 0.028×age (years) - 3.973×sex (men=1, women=2) + 0.097×weight (kg) + 0.148×height (cm) + 0.147×calf circumference (cm) - 8.734. |
| Appendicular skeletal muscle mass index (ASMI, kg/m^2^) | Not applicable | ASMI = ASM / height in meters squared |
